# Supplementary material for: Context-dependent correlations mislead transcriptomic network inference in bulk and single-cell data
Source: bioRxiv. 2026 Jun 28:2026.06.23.733936. Preprint. [Version 1] doi: 10.64898/2026.06.23.733936 (PMC13320913; doi:10.64898/2026.06.23.733936)
Supplement: Supplement 1 [file media-1.pdf]

Additional file 1: Supplementary material

Context-dependent correlations mislead transcriptomic network inference in bulk and single-cell data

Amir Asiaee, Polina Bombina, Reginald L. McGee II, Jake Reed,
Zachary B. Abrams, Lynne V. Abruzzo, and Kevin R. Coombes

This file provides definitions of key terms, robustness and extension analyses, and extended notes for the Research article.

Definitions

Table S1 collects the working definitions used throughout the main text.

Table S1: Key terms used throughout the Research article and this supplement.

Term	Definition
Context	A partition of samples by a biological covariate (e.g., TCGA cancer cohort, GTEx tissue, single-cell cluster, BRCA molecular subtype) within which correlations are computed.
Global (pooled) correlation	Pearson correlation r_{global} computed across all samples, ignoring the context partition.
Within-context correlation	Pearson correlation r_c computed using only samples assigned to context c.
Strong pooled pair	A pair with $\lvert r_{\text{global}}\rvert \geq 0.2$, used as the default effect-size threshold for reporting reversal rates.
Mixed-sign pair	A pair for which the set $\{r_c\}$ contains both signs after applying the sign tolerance ε.
Simpson reversal	A pair for which $\text{sign}(r_{\text{global}})$ disagrees with the within-context majority sign at tolerance ε.
Simpson-reversal rate	Fraction of strong pooled pairs that show a Simpson reversal at a given ε.
Mean-residualization	Subtraction of context-specific means from each variable before correlation; the resulting Pearson r_{resid} isolates within-context covariation.
Heterogeneity (Q, I^2)	Cochran's Q and Higgins–Thompson I^2 [1] computed on Fisher-Z–transformed r_c values to quantify dispersion across contexts.
High-variance domain	Subset of variables retained by variance filtering before pair enumeration (e.g., top 5,000 mRNAs $\times$ top 200 miRNAs in TCGA).
Sign tolerance (ε)	A threshold $\lvert r_c\rvert < \varepsilon$ that treats a within-context correlation as effectively zero when computing majority sign and mixed-sign status.

Supplementary figures

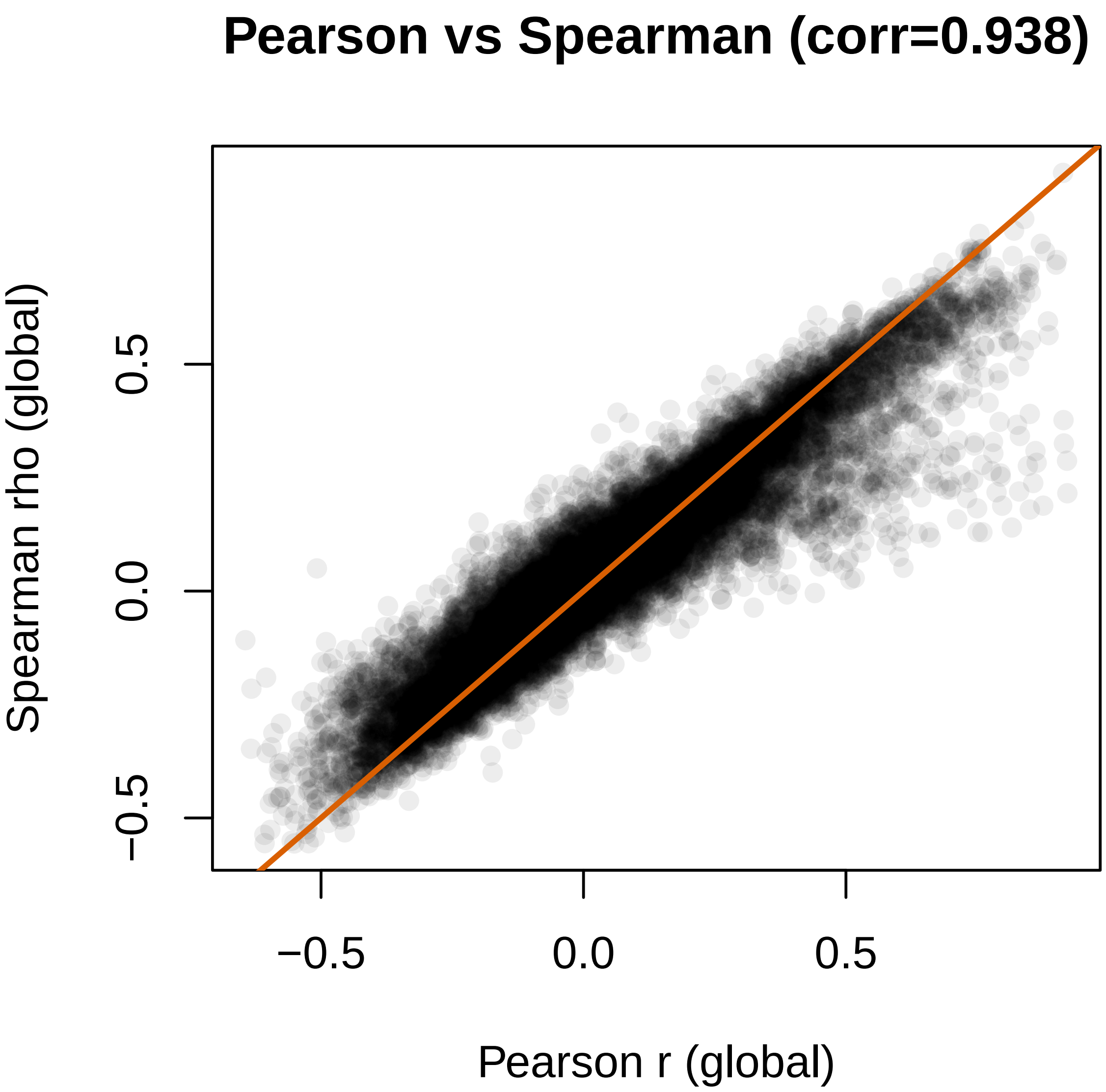

Figure S1: Pearson versus Spearman robustness in TCGA. On a 50,000-pair high-variance subset (1,000 mRNAs × 50 miRNAs), per-pair Pearson–Spearman agreement averages 0.94 across cohorts. The Simpson-reversal rate at $|r_{\text{global}}| \geq 0.2$ is 12.6% under Spearman versus 13.3% under Pearson, so the qualitative picture does not depend on the correlation measure.

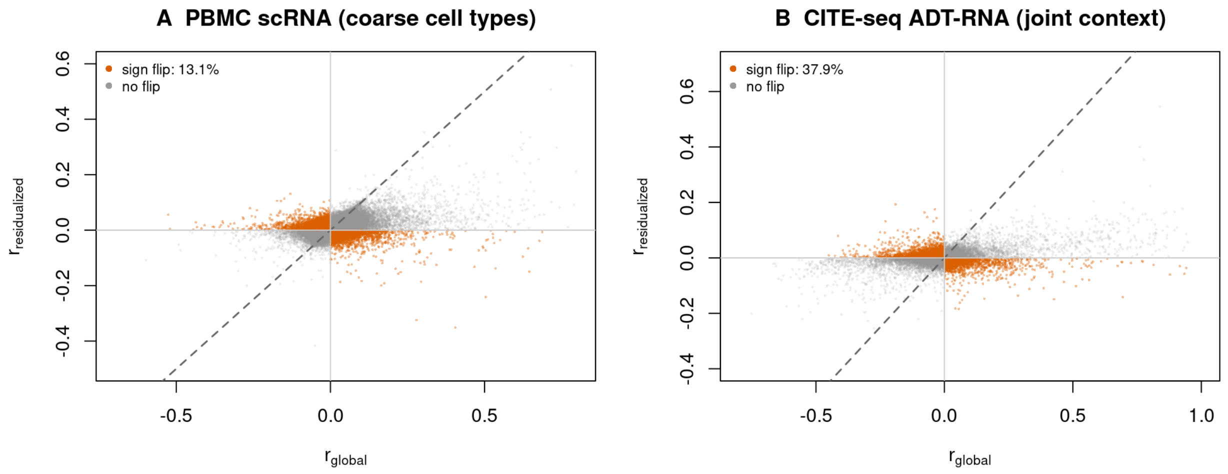

Figure S2: Single-cell global versus residualized correlations across modalities. Each point is a gene–gene pair (PBMC scRNA-seq) or protein–RNA pair (CITE-seq surface protein measured by antibody-derived tag, ADT); $x = r_{\text{global}}$, $y = r_{\text{resid}}$ after cell-type-mean removal. Pairs with $\text{sign}(r_{\text{global}}) \neq \text{sign}(r_{\text{resid}})$ are colored. The diagonal band of flipped pairs at large $|r_{\text{global}}|$ shows that strong pooled associations are not protected from reversal once context is removed.

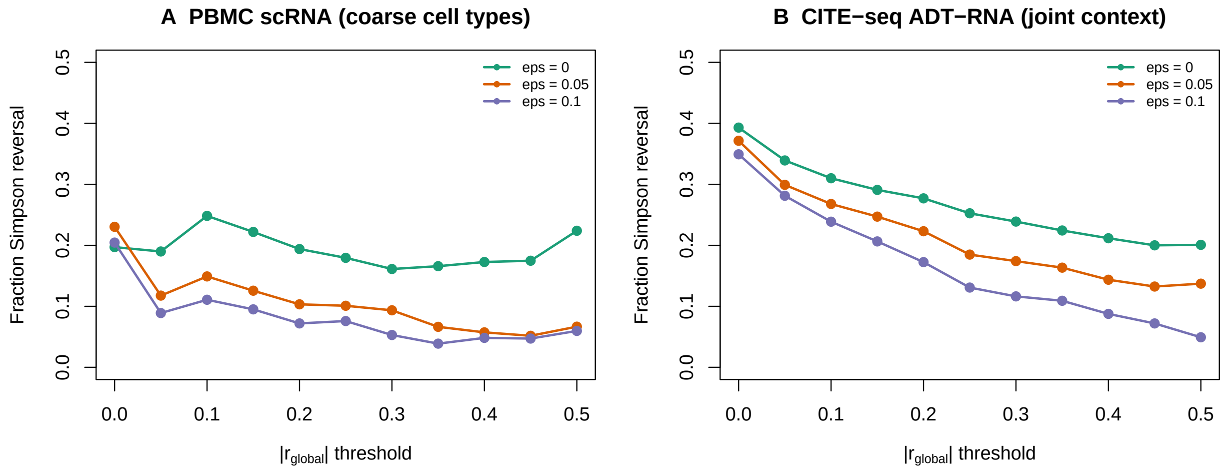

Figure S3: T-cell subtype refinement reduces reversal in PBMC scRNA-seq. Simpson-reversal rate versus effect-size threshold $|r_{\text{global}}|$ for coarse cell types (T, B, NK, monocytes, dendritic, platelets) versus T-cell subtypes (CD4 naive, CD4 memory, CD8, regulatory). At $|r_{\text{global}}| \geq 0.05$ the rate drops from 11.8% to 0.13%; at $|r_{\text{global}}| \geq 0.2$ it drops from 10.3% to 0% (0 of 41 eligible pairs).

Supplementary notes

Robustness to the correlation measure

The TCGA results are quantified with Pearson r, but rank correlation gives the same picture. On a high-variance subset of 50,000 miRNA–mRNA pairs (1,000 mRNAs $\times$ 50 miRNAs), Pearson and Spearman r agree pair-by-pair with mean concordance 0.94 across the 31 cohorts. The Simpson-reversal rate at $|r_{\text{global}}| \geq 0.2$ is 12.6% under Spearman versus 13.3% under Pearson (Fig. S1). Reversal and mixed-sign behavior are properties of the contextual data, not artefacts of distributional assumptions in r.

Refining cell-type context within PBMCs

Coarse cell types in the PBMC 3k dataset give a 10.3% Simpson-reversal rate at $|r_{\text{global}}| \geq 0.2$. Re-partitioning the T-cell compartment into CD4 naive, CD4 memory, CD8, and regulatory subtypes drops the rate to 0% on the 41 pairs that remain eligible, and from 11.8% to 0.13% at the more permissive $|r_{\text{global}}| \geq 0.05$ threshold (Fig. S3). The same compositional mechanism described in the main text – between-group mean shifts dominating pooled correlations – is what finer partitions absorb: once the dominant mean shift is between T-cell subtypes rather than between T cells and monocytes, the within-context correlations become consistent in sign.

Rare stable associations

A small minority of pairs are stably signed across every cohort in which they are measurable. In TCGA, 6,413 of the 692,770 measurable miRTarBase v10 [2] pairs (0.9%) are uniformly negative across cohorts, and the analogous uniformly-signed fraction among strong-effect pairs is well under 1%. These stable pairs include canonical tissue-restricted relationships such as miR-122 [3] within hepatic-lineage cohorts. Their existence confirms that the analysis can detect consistency when it is present, and it sharpens rather than softens the contrast with the heterogeneous majority.

Extended methods

Full data-source, preprocessing, correlation, heterogeneity, Simpson-decomposition, mixed-sign, and BRCA-subtype specifications are given in the main-text Methods section. All reproducibility scripts and the "correlation in context" R interface, including the random-effects meta-analytic estimator [4], the Fisher Z transform [5], I^2 heterogeneity [1], and Benjamini–Hochberg FDR control [6], are available at `https://github.com/AsiaeeLab/context-corr`.

References

[1] Julian P. T. Higgins and Simon G. Thompson. Quantifying heterogeneity in a meta-analysis. *Statistics in Medicine*, 21(11):1539–1558, 2002. doi: 10.1002/sim.1186.

[2] Shidong Cui, Sicong Yu, Hsi-Yuan Huang, Yang-Chi-Dung Lin, Yixian Huang, Bojian Zhang, Jihan Xiao, Huali Zuo, Jiayi Wang, Zhuoran Li, et al. miRTarBase 2025: updates to the collection of experimentally validated microRNA–target interactions. *Nucleic acids research*, 53 (D1):D147–D156, 2025. doi: 10.1093/nar/gkae1072.

[3] Simonetta Bandiera, Sébastien Pfeffer, Thomas F Baumert, and Mirjam B Zeisel. miR-122 – a key factor and therapeutic target in liver disease. *Journal of hepatology*, 62(2):448–457, 2015. doi: 10.1016/j.jhep.2014.10.004.

[4] Rebecca DerSimonian and Nan Laird. Meta-analysis in clinical trials. *Controlled Clinical Trials*, 7(3):177–188, 1986. doi: 10.1016/0197-2456(86)90046-2.

[5] Harold Hotelling. New light on the correlation coefficient and its transforms. *Journal of the Royal Statistical Society: Series B*, 15(2):193–225, 1953. doi: 10.1111/j.2517-6161.1953.tb00135.x.

[6] Yoav Benjamini and Yosef Hochberg. Controlling the false discovery rate: a practical and powerful approach to multiple testing. *Journal of the Royal Statistical Society Series B: Statistical Methodology*, 57(1):289–300, 1995. doi: 10.1111/j.2517-6161.1995.tb02031.x.
